# The New South Wales Allied Health Workplace Learning Study: barriers and enablers to learning in the workplace

**DOI:** 10.1186/1472-6963-14-134

**Published:** 2014-03-25

**Authors:** Bradley Lloyd, Daniella Pfeiffer, Jacqueline Dominish, Gaynor Heading, David Schmidt, Annie McCluskey

**Affiliations:** 1 Health Education and Training Institute, NSW Health, Gladesville, NSW, Australia; 2 Centre for Education and Workforce Development, Sydney Local Health District, Rozelle, NSW, Australia; 3 Discipline of Occupational Therapy, Faculty of Health Sciences, The University of Sydney, Lidcombe, NSW, Australia

**Keywords:** Workplace learning, Education, Allied health, Continuing professional development

## Abstract

**Background:**

Workplace learning refers to continuing professional development that is stimulated by and occurs through participation in workplace activities. Workplace learning is essential for staff development and high quality clinical care. The purpose of this study was to explore the barriers to and enablers of workplace learning for allied health professionals within NSW Health.

**Methods:**

A qualitative study was conducted with a purposively selected maximum variation sample (n = 46) including 19 managers, 19 clinicians and eight educators from 10 allied health professions. Seven semi-structured interviews and nine focus groups were audio-recorded and transcribed. The ‘framework approach’ was used to guide the interviews and analysis. Textual data were coded and charted using an evolving thematic framework.

**Results:**

Key enablers of workplace learning included having access to peers, expertise and ‘learning networks’, protected learning time, supportive management and positive staff attitudes. The absence of these key enablers including heavy workload and insufficient staffing were important barriers to workplace learning.

**Conclusion:**

Attention to these barriers and enablers may help organisations to more effectively optimise allied health workplace learning. Ultimately better workplace learning may lead to improved patient, staff and organisational outcomes.

## Background

Workplace learning involves ‘learning through participating at work’ ([1] p.210) and ‘learning that is stimulated by workplace activities’ ([2] p.119). Within allied health, workplace learning is largely an unfamiliar term. A similar concept that is more familiar to allied health professionals (AHPs) is continuing professional development (CPD); *‘…the means by which members of the profession maintain, improve and broaden their knowledge, expertise and competence, and develop the personal and professional qualities required throughout their professional lives.*’[3] The main difference between CPD and workplace learning is that the latter is focussed on learning within the workplace (where working and learning co-occur [4]), whereas CPD is a broader concept more commonly referring to external workshops and conferences (away from the workplace). The focus of this paper is workplace learning, which for the purpose of this study was defined as CPD that is stimulated by, and occurs through, participation in workplace activities.

AHPs work across a range of settings including acute and rehabilitation hospitals and community centres. Workplace learning can occur in all of these work sites, including on hospital wards, within treatment rooms, operating theatres, conference rooms, patient/client homes, offices, corridors, nurses’ stations and even in staff tea-rooms. Learning activities in these work sites can be intentional (structured and planned) such as a presentation in a hospital conference room (formal learning). Alternatively, learning may be unstructured and unplanned (informal learning) such as ad-hoc reflections with peers. A large part of workplace learning also occurs incidentally without the employee being fully aware that learning is occurring [5]. Regardless of the nature of workplace learning, it can occur at the individual, team or department level, as well as at an organisational or system level [6].

Workplace learning has been reported to improve individual, team and organisational outcomes in the health setting [2], including job satisfaction [7]. In addition, this mode of professional learning is thought to be a cost efficient approach for achieving health organisational goals [2]. A ‘value for money’ learning approach is important given the current fiscal constraints facing many health systems. Moreover, learning which occurs through engaging in workplace activities suggests potential for developing skills and knowledge specific to organisational and patient/client needs. Identifying factors that restrict or facilitate workplace learning (barriers and enablers) may improve workplace learning by enabling more targeted education and training strategies. There has been little research in this area specific to allied health. In medicine and nursing Manley and colleagues [2] found that having an organisation-wide learning philosophy and supportive organisation-wide infrastructure were key enablers of workplace learning. Support for learning, as well as having access to appropriate technology for learning were enablers in another study involving managers from a range of businesses [8]. Common barriers to workplace learning have included lack of time [8,9], a negative workplace culture, an absence of challenging work tasks, lack of expert support and advice, absence of expertise, ‘opaque knowledge’ (knowledge remote to the learner/task) and limitations of instructional media [10]. Of those studies on AHPs in this area known to the authors [11-13], research has focused on CPD rather than workplace learning, and has been limited to physiotherapists. Therefore this study was designed to identify and explore barriers to, and enablers of, workplace learning for AHPs within the study setting. The study also aimed to explore understanding and experiences of workplace learning for this group. To our knowledge this was the first study to specifically explore the concept of workplace learning in an allied health setting.

## Methods

### Study design

Using the framework approach [14], a qualitative study was conducted. The framework approach was suitable for the relatively short timeframe and pre-determined aims and objectives of this study. Data were collected via semi-structured interviews and focus groups. Ethics approval was granted by the Sydney Local Health District Ethics Review Committee (Royal Prince Alfred Hospital Zone) (Protocol No X12-0210 & LNR/12/RPAH/336). Written informed consent was obtained from all participants.

### Sample and recruitment

New South Wales (NSW) is one of eight States and Territories of Australia, with a population of 7.3 million people [15]. NSW Health provides public health services to people across eight metropolitan and seven rural/regional health districts, as well as three specialty health networks. Within this setting purposive sampling was used to recruit a maximum variation sample [16]. The investigators purposively sought to include a range of AHPs from three stakeholder groups (clinicians, professional educators and managers), from multiple allied health professions, multiple employment sites and settings and with a variety of demographic characteristics (including age, sex, years of experience and part-time/full-time employment). The professional educator group comprised AHP staff employed by NSW Health to perform a dedicated role in allied health staff education (specific to one or multiple allied health disciplines). A survey carried out by the NSW Health Education and Training Institute in 2012 identified less than 50 of these staff working in NSW [unpublished data]. Each of the three stakeholder groups was identified as important and unique for addressing the aims of the study. As it is common for AHPs to perform dual roles (e.g. 50% clinical, 50% management responsibilities), staff were classified in this study according to their self-identified ‘primary role’.

Six health districts and networks were randomly selected using a stratified procedure to ensure a range of sites were included without selection bias. The remaining health districts and networks included resulted from purposive selection of key staff (key informants and professional educators) who were not from one of the six randomly selected sites. Invitations to express interest to participate were sent directly from the first author and/or via the directors of allied health or their representative via their local allied health email distribution list. The investigators purposively selected participants to include in each focus group from the sample of those willing and available to participate to obtain maximum variation.

### Data collection

Three key informant interviews were conducted individually with one allied health clinician, one allied health professional educator and one allied health manager. These participants were purposively selected by the investigators for their unique knowledge and insight into allied health education and training. Key informant interviews were designed to inform discussions in subsequent focus groups. Focus groups were held separately for clinicians, managers and educators. Interviews were approximately 60 minutes and focus groups approximately 90 minutes in duration.

A basic definition of workplace learning was provided, with examples, at the beginning of each interview and focus group. However, participants were not restricted in their interpretation of workplace learning as it was one of the aims of the study to explore AHPs understanding of workplace learning. Initial questions explored participants’ background and knowledge, their experiences of, and attitudes toward, workplace learning. Subsequent questions focused on barriers to, and enablers of, workplace learning. Questions were adjusted for each target group (i.e. clinician, manager, educator) and were emailed to participants in advance to provide an opportunity for reflection and promote efficient use of time in focus groups.

All focus groups and interviews were moderated by the first author (BL) with assistance from another team member (DP), who also acted as an ‘observer’ and took hand-written notes. Following each focus group BL and DP reflected on key issues discussed in the group and compared these with issues noted in previous groups. These discussions informed future interviews and focus groups and promoted reliability of data collection. Focus groups and interviews were held during work hours at the researcher’s office, or at the participants’ place of employment. Tele/videoconferencing facilities were used to enable participation of 10 participants who could not attend in person. Audio recordings of focus groups and interviews were transcribed verbatim by the first author (BL) or a private transcription provider. A copy of the transcript or a summary document was provided to participants after each interview and focus group to verify data.

Guided by the framework approach, the total number of interviews and focus groups was determined by the study timeframe and resources, and consideration of whether pre-determined aims were satisfied. The investigators did not plan to achieve theoretical data saturation. At the time of writing the study protocol, the investigators planned three ‘clinician focus groups’, three ‘manager focus groups’, and two ‘educator focus groups’. Four additional interviews and one extra focus group were conducted to include additional key stakeholders, including clinical specialists and executive directors of allied health.

### Data analysis

The Framework Approach [14] was used to guide data analysis. First, interviews were downloaded, transcribed, read and themes identified. Next, a framework was developed with key concepts and themes. The third step involved systematically coding data using the thematic framework. Data were then transferred into tables with key themes and sub-themes to build a visual representation of the data as a whole. During this process, the range of attitudes and experiences, including deviant cases for each theme were considered. The final step involved mapping and interpreting the tabulated data to further refine concepts, map the range and nature of phenomena, and find associations between themes. Data analysis commenced after the first key informant interview and was performed by the first author (BL). Peer checking occurred at regular intervals. For example, BL met with AM and GH to discuss key themes emerging after coding the first two transcripts. The first two transcripts and a subsequent focus group were also coded by another investigator (AM or DP) to promote trustworthiness. Consensus was reached between study investigators. All data were coded manually. Originally over 50 themes relating to barriers and enablers of workplace learning were identified, which then went through several ‘iterations’ of refinement. Data analysis often occurred across multiple steps at any one time.

**Figure 1:**
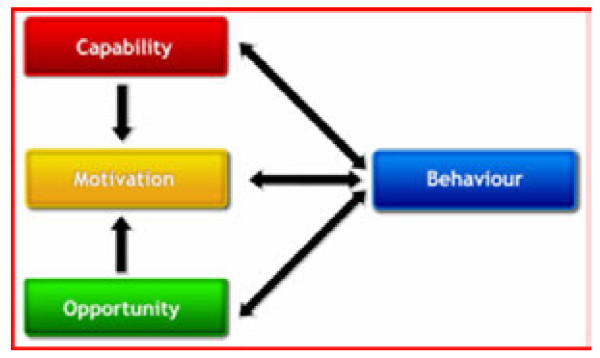
**The COM-B System – a model for understanding behaviour (**[17]** p.4).**

The ‘COM-B system’ [17] (Figure 1), a model for understanding behaviour, was selected to help interpret the data and explore mechanisms through which barriers and enablers exerted an effect on the behaviour of engaging in (and implicit outcomes of learning through) workplace learning. As the model depicts, behaviour is a product of the interaction between three components; Capability, Motivation and Opportunity. The model implies that changes to these components will influence behaviour (in this case, workplace learning), and that resultant changes to behaviour may in turn affect these components. It was thought using this model may assist with the design of subsequent implementation strategies to address the identified barriers to, and enablers of, workplace learning for AHPs.

Based on this model, the barriers to and enablers of workplace learning were explored as factors that restricted or that facilitated respectively 1) opportunities for AHPs to engage in workplace learning, 2) motivation for AHPs to engage in workplace learning, and 3) capabilities of AHPs to engage in workplace learning. For the purpose of this study ‘capability’ included relevant attributes, skills, knowledge, experience, and understanding of individual AHPs [6] and teams, as well as attributes of organisational systems and structures.

## Results

### Characteristics of the study sample

Expressions of interest were received from 129 AHPs from the 10 (of 18) health districts and networks included. Forty-six of these 129 participants were purposively selected and interviewed individually (n = 7), or in a focus group (n = 39).

A broad mix of participant characteristics was included (Table 1). The sample included 19 allied health clinicians, 19 managers of AHPs and eight educators. Many participants had dual roles, with most self-identified managers also having a clinical role. Ten allied health disciplines were represented. The disciplines with higher numbers of participants included physiotherapy (n = 9), occupational therapy (n = 9) and social work (n = 8). Seventy-two per cent (n = 33) of the sample were employed in a metropolitan health district or network and 28% (n = 13) in a rural or regional district. Within these settings staff were employed across acute and rehabilitation hospitals and community centres. Staff had a range of experience in working in the health system, from new graduate staff (< 1 year of experience) to senior executive staff (> 20 years of experience).

**Table 1:** Participant characteristics (n = 46)

**Characteristic**	**n**	**(%)**
Managers	19	(41%)
Head of Discipline/Service manager	13	
Allied Health Director^a^	5	
Other	1	
Clinicians	19	(41%)
‘Juniors’^b^	7	
‘Seniors’^c^	10	
‘Specialists’^d^	2	
Educators	8	(18%)
Discipline specific	5	
Allied Health	3	
Discipline		
Physiotherapy	9	
Occupational Therapy	9	
Social Work	8	
Speech Pathology	7	
Nutrition and Dietetics	4	
Psychology	3	
Medical Radiation Sciences	2^e^	
Podiatry	1^e^	
Genetic Counselling	1^e^	
Pharmacy	1^e^	
Nursing^f^	1^e^	
Employment site		
Metropolitan	33	(72%)
Rural/regional	13	(28%)
Employment status		
Full-time	36	(78%)
Part-time^g^	10	(22%)
Age (years)		
<25	0	(0%)
25-44	24	(52%)
45-64	21	(46%)
≥65	1	(2%)
Gender		
Female	34	(74%)
Male	12	(26%)

^a^Allied Health Directors included 3 district/network directors of allied health and 2 district/network discipline specific directors.

^b^‘Junior’ clinicians included Level 1–2 AHPs and psychologists/clinical psychologists.

^c^‘Senior’ clinicians included Level 3–4 AHPs and senior psychologists/clinical psychologists.

^d^Clinical specialists included Level 6 AHPs who identified their primary role as ‘clinician’.

^e^To maintain participant anonymity professions with ≤ 2 participants will be described as ‘allied health’ when presenting quotes, rather than by their profession.

^f^For readability the sample will be referred to as ‘AHPs’, inclusive of 45 AHPs and 1 nurse (the nurse was a manager of AHPs).

^g^Part-time employment was any paid employment that was self-identified to be ‘part-time’.

### AHPs understanding and experience of workplace learning

Most AHPs were unfamiliar with the term workplace learning and more familiar with terms such as professional development or CPD. Senior and experienced AHPs tended to have a more comprehensive understanding, capturing the incidental and informal qualities of workplace learning:

*“So essentially it’s learning that takes place at work and through one’s work … So it’s actually seeing the workplace as the classroom*”. Educator, Social Work

*“… it’s learning that’s triggered off from … experience that they’ve gathered or from challenges that they might have found in the workplace and … questions that arise from practice. Looking at the way they’re doing things and that type of thing”*. Manager, Nutrition and Dietetics (Rural/Regional)

Junior staff focused on more intentional and formal workplace learning activities:

*“… when I read ‘workplace learning’, I think straight away of all the in-services we have in our physio department and within the rehab department and all evidence-based practice; you know articles we go through as a team and all that sort of learning …”* Clinician, Physiotherapy

Interestingly, it appeared these lesser experienced AHPs and a few more senior AHPs did not fully appreciate the informal and incidental nature of much workplace learning, and only recognised intentional learning activities such as formal courses, conferences and workshops as contributing to their professional development. Workplace learning was often understood by these AHPs as something separate from practice and patient care:

*“… trying to still make that 85% clinical time and you’ve got 15% to do all your admin, your stats, and then you’ve got your learning on top of that …”* Clinician, Speech Pathology

Despite differences among participants in their familiarity with the term ‘workplace learning’ and their interpretations of this concept, many examples of formal and informal workplace learning were provided (Table 2). Formal learning included on-site workshops, in-services and presentations. Informal learning included talking to peers, engaging in self-reflection and online forums. Examples of both individual and team (discipline specific and inter-professional) workplace learning were also provided. Little mention was made of broader organisational workplace learning.

**Table 2:** Examples of workplace learning activities described by allied health professionals

	Mandatory training (e.g. fire safety, infection control)
More formal*	In-services/presentations/grand rounds
	Workshops
	Orientation
	Research/quality improvement projects
	Audit and service reviews
	Benchmarking with other services
	Skill competency assessments
	Video/tele-health sessions/‘webinars’
	Developing clinical practice guidelines
	Literature/internet searches
	Performance management/goal setting interviews
	Journal club (and reading journal articles)
	‘Continuing education sessions’
	Complex case discussion meetings
	‘Master classes’
	Case reviews
	Peer review and feedback
	‘Supervision sessions’
	Simulation activities
	Case conferences
	Managing/supervising/mentoring staff
	Inter-professional practice
	Treating/assessing patients/clients
	Observing peers
	Reflective practice
	Brainstorming
Less formal*	Online forum/discussion groups
	Team discussion/talking to peers

*Note: workplace learning activities may occur in less/more formal situations dependent upon the exact nature and specific setting in which these activities are completed.

Overall, AHPs in this study appeared to be engaging in a range of both formal and informal workplace learning activities. They acknowledged that workplace learning included learning through formal events such as on-site presentations and workshops, and informal learning through discussions with colleagues and patients. A belief that workplace learning only occurred formally, off-site and separate to practice was evident in some instances, in particular for less experienced junior staff.

### Factors that enabled or limited workplace learning

**Figure 2:**
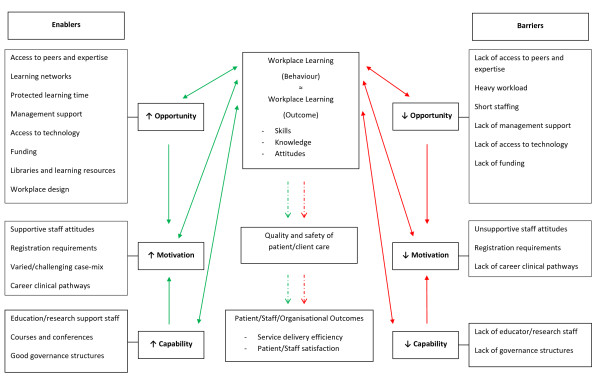
Concept map of barriers to and enablers of workplace learning for allied health professionals.

The key enablers of workplace learning described most frequently by the AHPs in this study included having access to peers, expertise and ‘learning networks’, protected learning time, management and organisational support for learning and positive staff attitudes. The absence of these enablers, in particular a heavy workload, insufficient staffing and lack of access to peers and expert knowledge were key barriers. These factors and other important barriers/enablers reported by the group were illustrated in a concept map incorporating the COM-B model (Figure 2). As noted earlier, this model of behaviour was used to help explore the mechanisms through which the identified barriers and enablers influenced workplace learning. The concept map additionally aimed to contextualise the data collected to organisational implications described by participants and in the literature such as the quality of patient care and staff satisfaction (represented by dashed lines in the figure). Barriers and enablers were grouped in the figure according to the component of the COM-B model (Capability, Opportunity or Motivation) by which the effect on AHPs’ workplace learning was determined to be most prominent. The components of the COM-B model will be used in this section as a framework to present the findings.

### Opportunities for AHPs to engage in workplace learning

Key factors that enabled or limited workplace learning through promoting or restricting opportunities to engage in workplace learning (‘opportunity factors’) included access to peers, expertise and ‘learning networks’, protected learning time, and management and organisational support. Other important but less commonly reported opportunity factors included access to technology and funding to support learning, libraries and learning resources and workplace design.

#### Access to peers, expertise and ‘learning networks’

The strongest factor that influenced the opportunities available to AHPs to engage in workplace learning was access to peers and expertise. AHPs frequently described that observing, interacting with and simply ‘being around’ experienced clinicians or ‘experts’ provided important opportunities for workplace learning:

*“… for instance in intensive care, I am exposed to minds that are just unbelievable … these are people that you think thank god they’re looking after those patients, they’ve got brilliant minds … even by just absorbing [and] being around those conversations I learn a lot”.* Educator, Social Work

*“… all the physios are usually in the gym at the same time so I can watch what my senior’s doing with her patients and learn just by watching. And … they can watch what I’m doing so … if I’m doing something that’s not working quite well … they’re right there to ask, this isn’t working, what should I do?”* Clinician, Physiotherapy

Having access to peers from the same discipline as well as from other disciplines (inter-professional practice) facilitated workplace learning:

*“… sometimes during joint work as well … you learn a lot from doing multidisciplinary clinics or joint work with groups or things like that. Because you’re seeing your colleagues in practice, and so that actually creates a lot of [workplace] learning”* Educator, Allied Health

*“Every fortnight we have an orthopaedic registrar that comes … and through the process of when they're actually reviewing the patients, they’ll do a bit of … professional development, quite informal, but [we] will learn a lot of techniques, [we] will learn a lot of the orthopaedic tests.”* Manager, Physiotherapy

Moreover, having a larger department with access to more peers on site, regardless of experience or seniority, also enabled workplace learning. Larger departments with more staff were reported to stimulate discussion and enable access to a greater breadth of knowledge:

*“… we have more access to workplace learning in that there’s more people around so we do have in-services and we do have case study reviews, and we can learn a little bit more from colleagues as far as case conferences and more senior allied health people”* Clinician, Physiotherapy (Rural/Regional)

However, some senior AHPs and those working in speciality areas reported that opportunities to engage in workplace learning were limited due to difficulty accessing colleagues with the level of knowledge or skills needed to facilitate their learning:

*“… being a senior therapist, there’s hardly any access to other senior therapists with more experience or more knowledge, so it’s difficult that way”.* Clinician, Physiotherapy

*“… none of my colleagues within the hospital are within my speciality. So I can’t really turn to any of them. I’d have to turn to external dietitians and there’s only a couple in New South Wales”.* Clinician, Nutrition and Dietetics

Staff in smaller departments/organisations and sole practitioner positions commented on the lack of peers and expertise in the workplace. This was particularly evident for staff working in rural/regional sites who described feelings of isolation:

*“… there is a degree of isolation I suppose. The problem is we don’t have really a lot of, very many hugely experienced clinicians in the department”.* Clinician, Physiotherapy (Rural/Regional)

*“I think another thing that … really is a problem for someone in a rural place like XX [regional site] is [that there is] no-one really with the clinical expertise in your field so there’s no clinical physio manager or clinical expert in physio for us to go to. We don’t know who our go to person is, it’s sort of an informal arrangement … you don’t know where your specialists are”.* Clinician, Physiotherapy (Rural/Regional)

However, there was evidence that using technology or organising ‘shadow placement’ (secondment) opportunities could help overcome this barrier and facilitate learning opportunities for this group by linking staff with peers from other workplaces:

*“We do a weekly teleconference where all the staff either have to present one case or give an overview of all the patients they're working with … it’s an opportunity to review how people are working with patients, get ideas from other staff, support, et cetera, particularly for rural staff who are isolated sole workers. So, it’s an opportunity for them to link in with other staff in the service”.* Manager, Allied Health

*“… sole physios or physios in rural areas being allowed the time … to go and … work in one of the larger hospitals in an area of either clinical need or something that they have an interest in … I think that would be a good way to have both some professional support so you get to go and meet the physios that work in those other areas, and update clinical knowledge as well. ‘Cause I think working on your own, you need contact with other professionals, whether that be the experts or just physios in general”.* Clinician, Physiotherapy (Rural/Regional)

The presence of established networks (‘learning networks’) enabled workplace learning through facilitating access to peers and expertise and allowing opportunity for peer review, clinical supervision and feedback. Networks were described for discipline-specific allied health managers and clinicians as well as inter-professional networks among staff sharing common clinical specialities (e.g. HIV, Paediatrics). There were examples of networks between sites within a health district, networks across health districts, networks outside NSW Health with private sector AHPs and networks with national and international membership.

There was little mention of the role that students and new-graduate staff played in workplace learning. However, a few AHPs did report that students and new-graduate staff facilitated workplace learning through promoting reflective practice:

*“… if you’re teaching a group of students and they bring up questions, that actually enables you to question your own clinical practice as well …”* Educator, Occupational Therapy

In summary, a key determinant of the opportunities provided for the learning of these AHPs was access to peers and expertise. Networks were also important as they facilitated communication and learning between AHPs. Those staff who worked in areas where there was greater access to peers, and in particular to highly experienced and knowledgeable peers, had more opportunities for learning than those staff at smaller sites without these links. Access to peers and experts facilitated learning through inviting discussion and allowing greater opportunity for observing and being guided by others.

#### Protected learning time

Another key determinant of the opportunity for AHPs to engage in workplace learning was the amount of dedicated or ‘protected’ time away from direct patient or client contact. AHPs stressed the importance of the need for time to think and reflect for quality learning, activities which were facilitated with protected time away from patients and administration:

*“… [the] capacity to have time away from direct client contact … for either more formal workplace learning or informal discussions, access to relevant literature, a whole range of different learning activities”.* Educator, Allied Health

The main examples of protected learning time described by these AHPs included time dedicated to team in-service presentations, case study reviews and clinical supervision:

*“The other thing that we did was just protect some time that we would have [an] in-service presentation within the therapy team”.* Clinician, Occupational Therapy

Rarely was there any mention of protected time for individual reflection and study. A heavy clinical or administrative workload often competed for their time and restricted opportunities for workplace learning. There was a clear expectation that AHPs would meet demanding clinical service needs at the cost of protecting time for learning:

*“… we’re constantly being harassed to see if we can get people out of hospital [when] … it wasn’t planned that they leave quite so soon. So this … pressure on us … is compromising the time you have for learning”.* Educator, Occupational Therapy

Administrative workloads took time away from engaging in learning activities, with AHPs reporting considerable time spent performing client bookings, managing recruitment processes and completing other tasks such as photocopying. This was particularly evident for senior and management staff:

*“… trying to recruit someone takes nearly half your life by the time you put it all into the recruitment system, and then you try and work out how to schedule people in for an interview and … something’s got to give when all these other expectations are just growing and growing”.* Manager, Speech Pathology

Understaffing was another common complaint that limited learning opportunities. Some AHPs reported that their team was understaffed due to difficulties in recruitment and no back-fill for staff on leave. The additional workload for AHPs to cover this short-staffing took time away from workplace learning. This was clearly visible for the department this psychologist worked in:

*“… how short of staff we have been over the last year with other staff having to cover for the gaps within the service and the recruitment process taking too long, like up to nine months to recruit someone into a position, which means the rest of the team on that particular unit and of that particular profession ends up having to cover for that. That makes it very hard, and especially when you take away the allied health manager position and then the seniors are stretched up, stretched down. The staff are stretched up and stretched across”.* Clinician, Psychology

Overall, it was clear that some AHPs in this study were restricted in their opportunities for workplace learning by the lack of protected learning time. The time for learning for these AHPs was taken away by heavy clinical and administrative workloads, and this time was further limited in some departments due to under-staffing. Workplaces which protected time for intentional learning activities such as journal clubs, in-services and formal clinical supervision afforded greater opportunities for workplace learning to occur.

#### Management/organisational support

The level of management and organisational support for learning had a strong influence on the workplace learning opportunities available for AHPs. Examples of management support that afforded opportunities for learning included organising and encouraging workplace learning activities, protecting time for learning and allocating ‘challenging tasks’. Moreover, organisations supportive of learning promoted a culture of workplace learning, such that AHPs felt that it was ‘ok’ to engage in, and even lead, workplace learning:

*“It’s vital that managers are behind you because it’s very hard for allied health to seek out and justify it, or explain the time, or make the time to do workplace learning, if they don’t have that support from above”.* Educator, Allied Health (Rural/Regional)

*“… [my manager] has been very supportive … in my own development … [by] challenging me a little bit in terms of the tasks that she gives me to do …”* Clinician, Physiotherapy (Rural/Regional)

Learning opportunities were limited for several rural/regional AHPs because they did not feel well supported by management to undertake workplace learning. They reported there was a lack of encouragement and support for group discussions, and time for reflection from management:

*“We don’t have any allied health head. There’s no senior OT [occupational therapist]. We’re … just two part-timers, equivalent to each other, and our manager [doesn’t] … understand what OTs [occupational therapists] do in their day to day work … there’s no encouragement from anyone else to participate … in workplace learning”.* Clinician, Occupational Therapy (Rural/Regional)

Less often, there were also examples where a lack of management support restricted workplace learning for AHPs working in metropolitan hospitals:

*“… I haven't felt particularly supported by the upper management. … I haven't felt strong support from the management to develop better skills in something”.* Clinician, Psychology

Further, one AHP noted that their organisation did not offer formal learning opportunities to AHPs:

*“I think you don’t really have to look far to see that that's the exact direction of the organisation, because we have a calendar where we have the learning and development group that put out all the courses that are available in six months, and if you look at it, there's not a single thing on there for allied health”.* Manager, Physiotherapy

In sum, the level of management and organisational support for learning acted as a key barrier or enabler for workplace learning through influencing the amount and quality of learning opportunities available to staff. Those workplaces where managers carefully considered allocation of work tasks, scheduled protected time for learning activities and promoted a culture of learning provided greater opportunities for AHPs to learn.

#### Other ‘opportunity factors’ for workplace learning

Other factors that affected the opportunities available for AHPs to engage in workplace learning that were important but less frequently described included access to appropriate technology, funding, libraries and learning resources and workplace design.

The access to widely available technology, including computers, internet and email and tele/videoconferencing facilities, enabled or restricted the opportunities available for staff to learn. Technology enabled efficient access to literature, facilitated peer discussion and participation in ‘webinars’, through which AHPs could develop their knowledge and skills:

*“… they do have their own computer, so they have their own email address … everybody has access to the internet and that allows them to do things like webinars and it allows them to get into … whatever search engine they want to - to look up the literature, it allows them to email people who are acknowledged in the profession as experts and ask them advice … There’s a … chat group and people will email in with problems and … I know that a couple of my members of staff are often emailing back responses to how you manage this problem … I think that it does create this culture of inquiry and a culture of excitement around learning and teaching”* Manager, Nutrition and Dietetics

Technology was especially important for rural AHPs because it could be used to gain access to peers and expert knowledge which were often missing at these sites:

*“I think the advent of webinars and … videoconferencing links and all of that certainly make it easier for rural based practitioners to engage in ongoing learning”.* Director of Allied Health (Rural/Regional)

Limited access to appropriate technology restricted opportunities for AHPs to engage in workplace learning. Some AHPs reported difficulty in delivering education due to internal firewalls, while others could not participate in videoconferences due to a lack of equipment.

*“… one of our frustrations with providing education through technology is that each of the Local Health Districts has different IT teams, and with that comes different firewalls … we've tried to use webinars and things like that, and a lot of our initiatives have been challenging for some areas because of the internal firewall … that's frustrating … [for] many clinicians trying to access education …”* Educator, Allied Health (Rural/Regional)

Opportunities for workplace learning were also influenced by the level of funding dedicated to support learning. There was mention by some AHPs of little or no funding to attend courses and conferences, for back-filling of staff on leave, for more dedicated education and research support positions, and for some innovative workplace learning strategies (e.g. development of e-learning packages):

*“… there’s not the money to do the training and to keep you up to date clinically, and the money to keep you up to date management wise”.* Manager, Speech Pathology (Rural/Regional)

Libraries and librarians also provided opportunities for workplace learning for some AHPs, enabling literature searches and access to journal articles:

*“I think one thing that actually helps … is that we’ve got quite a pro-active library and I know I get an email from them every time the journals that are relevant to me in my various interest areas – the new journal becomes available, so they tell me that that particular journal has arrived and they give me the link just to click on … that’s something that really does help when you’re time limited”.* Educator, Occupational Therapy

In addition, it was noted that centralising important learning resources, for example through internal ‘intranets’, specialty newsletters or websites could enable workplace learning through providing easier and more efficient access for AHPs to locate, share and use relevant resources:

*“… the central repository for all that type of information is always helpful, like a resource centre … We're in the process of developing our Intranet site and a part of that's allied health and I'm putting … hyperlinks to … sites where there's information they need to access”.* Manager, Physiotherapy

Designing workplaces to facilitate access to and discussion between peers enhanced opportunities for some AHPs to engage in workplace learning. This included having shared office spaces, and shared treatment and assessment areas. For example:

*“… our thing that makes it fairly easy [to engage in workplace learning] in terms of my specific team and the multidisciplinary team and the informal learning we have there is … the physical layout of our office where all the staff are in the one office, they are not broken up into individual offices so … the office environment is conducive to discussion and sharing … of knowledge …”* Manager, Allied Health

In this section several factors were described that could enable or restrict workplace learning for AHPs through enabling or restricting, respectively, the learning opportunities available to staff. Those factors most influential on workplace learning included access to peers, expertise and learning networks, protected learning time and management and organisational support for learning. Other factors that were also important but less commonly described included access to technology and funding to support learning, libraries and learning resources and workplace design.

### Motivators of workplace learning for AHPs

The key motivational factor influencing engagement in workplace learning that was most frequently reported by AHPs was staff attitudes toward learning. Other motivational factors described less frequently included mandatory CPD requirements as part of professional registration, varied and challenging patient/client case-mix and clinical pathways for career progression. Based on the COM-B model, the opportunity and capability factors discussed in this paper also influenced the level of motivation of staff to engage in workplace learning (Figure 2).

#### Staff attitudes and perceptions toward workplace learning

Allied health clinicians, educators and managers from all settings, rural and metropolitan, overwhelmingly stressed the importance of, and need for, workplace learning. These AHPs highly valued workplace learning as a means by which they ‘keep up-to-date’, ‘keep on track’, ‘put skills into practice’, ‘maintain competency’, ‘boost morale’, ‘identify specific needs’, become ‘more efficient with clients’ and ‘do a better job’. Some AHPs, predominantly managers, also reported that workplace learning was critical for providing ‘high level care to patients’, ‘optimising patient outcomes’, as well as for job satisfaction and staff retention:

*“[Workplace learning] … keeps us on track. It’s what we actually do. So we need to continue to be trained in what we’re doing”.* Clinician, Occupational Therapy

*“[Workplace learning is] … something we value very highly as a way of keeping ourselves up-to-date with the latest evidence-based practice [and] techniques and theories.”* Manager, Social Work

The absence of good workplace learning was described by one director of allied health as a potential threat to patient safety which could result in complaints:

*“… it just comes back to bite you in some other form if you don't do it [workplace learning] well, and you see that in the form of complaints, you see that in the form of incidents …”* Director of Allied Health

AHPs also directly reported that attitudes supportive of workplace learning facilitated learning:

*“… some other things that make it [workplace learning] easy are just personal attitudes … The staff that we’ve got are quite open to learning and development and recognise the importance of that and are also very keen to learn from each other as well”.* Manager, Allied Health

Some AHPs reported that their personal attitude and passion for their job and wanting to be ‘a good clinician’ and wanting ‘the best for clients/patients’ motivated them to engage in workplace learning. In response to the question “what motivates you to engage in workplace learning?” a new graduate occupational therapist replied:

*“I’m very passionate about the job I do and the … patients that I see. And I think that’s what keeps us going and keeps us motivated”.* Clinician, Occupational Therapy

Finally, one senior speech pathologist noted:

*“… it’s about doing things well and doing things the best that we can and wanting the best for our clients, as well as being good clinicians and … that drives us”.* Clinician, Speech Pathology

Negative or unhelpful staff attitudes prevailed in some workplaces, inhibiting the motivation of AHPs to engage in workplace learning. This included comments by some participants that learning occurred predominantly via formal courses and conferences, ignoring the learning opportunities within the workplace. There was also direct report by some staff of their peers not wanting to engage in workplace learning, ‘already knowing everything’.

*“… we’ve had trouble with peer supervision … some people feel they don’t want to be supervised”.* Manager, Occupational Therapy

*“… we’ve tried to get the physio [education] group up and running again this year, the informal group, but because some of the people had been there for a while, they’re really not interested”.* Clinician, Physiotherapy (Rural/Regional)

The nature of individual attitudes toward, and perceptions of, workplace learning was a key determinant of the degree to which AHPs were motivated to engage in workplace learning. Overall, most AHPs had positive and supportive attitudes toward learning at work, however negative or unhelpful attitudes were also evident in the workplaces of some AHPs.

#### Other motivational factors for workplace learning

Other factors that affected the motivation of staff to learn at work included professional registration requirements, the nature of patient/client case-mix and clinical pathways for career progression.

A recent motivating factor for some AHPs to engage in workplace learning was the introduction of mandatory CPD requirements for professional registration. These mandatory requirements stipulated the need for, and the value of workplace learning. For example these allied health managers noted:

*“I think national registration has made it easier for the registered professions, because there are now mandated amounts of continuing professional development they have to do. At some point the system’s … accepting that that has to be done”.* Manager, Medical Radiation Sciences (Rural/Regional)

*“I think the fact that the registration boards recognise things like reviewing journal articles and journal clubs … as part of your professional learning certainly makes it easier and more viable to participate in those sorts of activities when you can put it towards your CPE [continuing professional education] points for registration”.* Manager, Physiotherapy (Rural/Regional)

However, one allied health manager downplayed the effect of professional registration on workplace learning:

*“… [national registration] requirements haven’t really come into … planning of learning and continuing professional development at all. From what I gather from staff those requirements are fairly easy to achieve through a lot of informal learning, primarily, and the odd formal like external conference or course and things like that they’ll go to … so I don’t sit down and look at what AHPRA [Australian Health Practitioner Regulation Agency] need and work towards that because my understanding is that it’s something that … can be achieved without a lot of planning and effort anyway”.* Manager, Allied Health

Having a varied and challenging patient/client case-mix also motivated some AHPs to engage in workplace learning:

*“… another thing that … does drive, in my team, us to want to learn more is clients that don’t fit the mould. So when you have clients that aren’t presenting as expected or who aren’t progressing as expected, then it does make you … step back and look at the big picture and think, what else could I be doing here?”* Clinician, Speech Pathology

A barrier to workplace learning for some AHPs was a lack of clinical pathways for career progression and this limited their motivation to engage in workplace learning:

*“I think it sometimes helps to have a clinical pathway for clinicians and I think we struggle to do that because generally, positions are a set award in a set position, and even if you've been there for five, six years and developed, there's nowhere else for you to go. And certainly not everyone wants to get into management”.* Manager, Psychology

However, one AHP noted how one of the allied health awards may facilitate workplace learning:

*“… I think that the allied health award helps with the … provision of workplace learning. Within my team … I’ve talked to all staff that have personal regrades or have a … clinical specialist position in a particular area … I’ve had lots of discussions with them in regards to their role in … education and training of other members of the team”.* Manager, Speech Pathology (Rural/Regional)

The key determinant of how motivated staff were to engage in workplace learning was their attitudes toward and perceptions of learning. Those AHPs who valued and recognised the importance of ongoing learning to their job role (patient care) appeared to be more motivated to engage more whole-heartedly in workplace learning opportunities. Other factors that were also described by some AHPs that influenced their motivation to engage in workplace learning included professional registration requirements, nature of patient/client case-mix and clinical pathways available for career progression.

### Capabilities of AHPs to engage in workplace learning

The capability of AHPs to engage in workplace learning was enhanced or limited through the existence of dedicated allied health professional educators, participation in external courses and conferences and good governance structures. These ‘capability factors’ were less commonly described by AHPs than the key opportunity and motivational factors described earlier.

#### Professional educators and research support staff

Dedicated professional educator and research staff could support learning by enhancing the capability of AHPs to engage in workplace learning. Participants noted that these dedicated staff enabled learning through encouraging AHPs to learn, teaching AHPs how to educate others, providing feedback and guidance on practice, synthesising literature, as well as through the development and delivery of tailored education programs:

*“… what’s clear to me is where that position [allied health professional educator] has existed they certainly are able to progress more implementation around allied health education programs …”* Director of Allied Health

*“… it’s very tough for staff who are working clinically to keep abreast of the literature … there’s nothing worse than giving a busy clinician a pile of 20 articles saying, go away and read these … I think that’s an area where people often need support to be able to do that”.* Manager, Social Work

*“… we actually had research managers that could help us do all of those things and somebody that had a responsibility to help us … do the audits, enter the data and … then feed back to us the data about those things”.* Clinician, Physiotherapy

Allied health managers and educators noted there was a lack of dedicated education positions and described this as a barrier to workplace learning, limiting capabilities of AHPs to learn:

*“… that’s the challenge around not having dedicated educator positions that the clinical work gets the priority and other activities like research and education tend to fall a bit behind … because there are so few educator positions those activities decrease when staff shortages happen …”* Director of Allied Health

Interestingly, some allied health managers felt that the allied health workforce did not have the skills or experience required to effectively carry out professional education roles:

*“… the allied health educator role is quite new to the award and I think we need to look at how … we create those positions but also how … we start to build a workforce that could actually fill those positions, because I think we have a lot of good clinicians, we don’t necessarily have good clinicians that are good educators, and that’s a skill that we need to teach people as well. It’s not something we learn in our degree”.* Director of Allied Health

*“… none of us signed up to be teachers, particularly. So, you know, what is actually the quality of what we’re delivering internally in terms of that - in terms of its quality from a teaching adult learning point of view? That’s another skill in itself”.* Manager, Social Work (Rural/Regional)

It was evident from the discussions with participants that there was a shortage of dedicated allied health education and research support roles. Where these roles existed, the capability of AHPs to engage in workplace learning appeared to be greater than in those workplaces lacking these positions.

#### External courses and conferences

Attending formal education away from the workplace enabled workplace learning for AHPs through the collection of new knowledge and exposure to ‘different ways of thinking’ that was able to be shared with colleagues upon return to the workplace. One director of allied health commented:

*“I think what going … to conferences and to courses [does is that] it introduces differences and makes us question as a department or as a discipline or as Allied Health, is this the way we should always practice, what are different ways of practising or learning”.* Director of Allied Health

A clinician noted that through attending courses and conferences: *“… [you] facilitate your critique … and also your application of evidence-based learning…”* Clinician, Allied Health. Attending courses and conferences also enabled workplace learning through facilitating the development of learning networks:

*“If you’re going … to the external conferences then you may gather networks so other services might be doing things a little bit differently, and you can … take from their learnings … You contact them back up and you say … “We’re about to embark on this …. Can you give me more information about what you’ve done?””* Manager, Speech Pathology (Rural/Regional)

There was evidence that attending relevant courses and conferences away from the workplace could strengthen the capability of AHPs to engage in workplace learning. These formal intentional learning events provided opportunities for staff to collect new knowledge and experiences, and to build new learning networks that could be accessed and shared with other staff in the workplace.

#### Good governance structures

Another factor that affected the capability of AHPs to engage in workplace learning was the level of good governance structures around supporting learning. Participants noted that having systems and structures in place that supported workplace learning would enable them to set up and facilitate greater access to learning opportunities such as supervision, goal setting, learning networks, orientation, evidence-based practice, reflective practice and performance development:

*“… that whole process of performance appraisal, managing for improved performance, professional development plan, if time’s put into developing them and developing strategies to meet those goals, then it’s much easier to facilitate workplace learning”.* Educator, Allied Health (Rural/Regional)

*“… building in a system where some things are … acknowledged as being really important, that we need to continue to do those, irrespective of whether we’re short staffed”.* Clinician, Physiotherapy

*“I think it would be good if managers had some sort of KPI [key performance indicator] around what … learning opportunities their staff were having. I think … we should have a system that makes managers more accountable for giving consideration to their staff’s learning opportunities”.* Manager, Social Work (Rural/Regional)

The absence of good governance to support learning was particularly evident for AHPs from one rural/regional health district. Their workplaces lacked the structures to support workplace learning, including absence of structures to foster clinical networks, team learning and performance review. For example, one AHP from this health service said:

*“… there was no feedback whether I was doing a good job, a bad job, or an indifferent job, or what gaps I had, or where we were going from here. It didn’t exist …“* Clinician, Occupational Therapy

Although often not directly referred to by participants, it was evident from discussions that existence of good governance structures designed to support and promote learning was important for enhancing the capability of AHPs to learn at work.

Overall, the capability of AHPs to engage in workplace learning was affected by the presence or absence of dedicated allied health professional education and research support staff, by the opportunities for AHPs to attend external courses and conferences and by the governance structures in place to support learning.

## Discussion

The primary aim of this study was to identify and explore the barriers to, and enablers of, workplace learning specific to AHPs within a large state-wide public organisation, NSW Health. There were four key messages from this study. First, access to peers, expertise and learning networks was the most important enabler of workplace learning. Second, large clinical and administrative workloads were the primary barrier to workplace learning, due in part to limited protected time for reflection and learning. Third, the level of management and organisational support for workplace learning was a key determinant of the learning opportunities available to AHPs. Finally, staff attitudes and perceptions of workplace learning also played a key role in enabling or restricting workplace learning through affecting the motivation to learn.

### The importance of peers, expertise and learning networks

Having access to peers, expertise and learning networks was the strongest enabler of workplace learning for AHPs in this study. Learning was facilitated through these factors by observing others, receiving guidance and feedback and inviting discussion. This finding agrees with others who have reported that ‘most learning occurs in group contexts’ ([6] p.88) and through ‘…critical discussion and debate with … workplace colleagues’ ([2] p.119). Moreover, a lack of expertise has previously been reported as a key barrier to learning [10]. Having little or no access to peers and expertise, including a lack of clinical supervision, was a key barrier that restricted opportunities for AHPs in this study to learn at work. This was a common barrier for AHPs in isolated or small departments, in particular for AHPs employed in regional or rural locations. Where access to peers and experts was lacking, there was evidence that establishment of learning networks, using technology (e.g. webinars and videoconferencing), attending courses and conferences, inter-professional practice and where appropriate, undertaking shadow placements helped to minimise this barrier.

### Managing workloads and protecting learning time

A large and heavy workload was the most commonly reported barrier to emerge in this study. For some AHPs this barrier was caused by short-staffing. As a result, AHPs consistently reported ‘not having enough time’ for workplace learning due to high clinical and administrative demands. This finding is consistent with other studies that have repeatedly described ‘a lack of time’ as being a major barrier to workplace learning [2,8,9,11,12]. Importantly not having time to stop and reflect, either as an individual AHP or as part of an allied health team or the wider organisation has the potential to compromise the safety of patient care. As noted by Eraut ([18] p.261), when the pressure for productivity forces staff to perform tasks at a speed beyond what even proficient workers consider appropriate “…then quality falls, the level of risk is higher and job satisfaction plummets.” Where time was protected for learning opportunities such as in-services, case study reviews and clinical supervision, there were greater opportunities for learning. It is important to note however, that a challenging workload, that is not excessive, could also stimulate workplace learning through motivating AHPs to problem solve and to develop more efficient work processes. Therefore finding the ‘right balance’ is difficult and likely needs to be highly individualised and contextual. Studies exploring the proportion of AHP work time spent in direct patient care versus time spent in reflection and critical thinking do not yet exist. This is an area suggested for future research.

### Management and organisational support

In this study, as in others [2,6,8,11,13], management and organisational support appeared to be a key determinant of workplace learning. Managers were probably identified as important because of their key role in creating and shaping learning opportunities through the allocation and structuring of work (e.g. allocating varied and challenging tasks and building in time for reflection and other learning activities). Managers also provided leadership, role modelling and feedback. Through these activities managers could promote an organisation-wide learning philosophy that enabled workplace learning [2]. The absence of management support appeared to correspond with fewer opportunities for AHPs to develop their knowledge and skills. Therefore, educating and up-skilling AHP managers in ways to effectively support workplace learning appears critical to enhancing the learning opportunities available to staff. As highlighted by another author [18] ‘…of all the mechanisms used at organisational level to promote learning the most significant is likely to be the appointment and development of its managers’ (p.271).

### Staff attitudes and perceptions of workplace learning

The attitudes and perceptions of AHPs toward learning appeared to be key determinants of their motivation to learn at work. Those staff who valued workplace learning and perceived benefits for their professional development and work role seemed to be more motivated to engage in, and actively seek out, opportunities for learning. Where staff lacked motivation to engage in learning opportunities often there was evidence of unhelpful or negative attitudes toward learning.

Interestingly, few AHPs commented on the importance of everyday activities such as treating and assessing patients for workplace learning. Apart from some more senior AHPs, when referring to workplace learning participants tended to focus on more formal and intentional types of learning such as department in-services and courses and conferences rather than, for example, on reflective practice during/after delivery of patient care. This perception has been described as a ‘course culture’ [11] and may have limited the learning of some staff who were not fully recognising the learning opportunities available in everyday work activities. Manley and colleagues [2] noted that “…opportunities for learning from everyday work are often not recognised because they are taken for granted” (p.113). The everyday work of most AHPs in our study was to provide care to patients and/or support staff with this role, so this may explain why learning through the delivery of patient care was rarely mentioned. Moreover this perception may partly explain why a heavy workload was only ever described by AHPs in this study as a barrier to, and never described as an opportunity for, learning, for example through providing opportunities to develop skills in time management and clinical prioritisation. Interestingly, one factor that may have contributed to a course culture was the low number of mandatory CPD hours stipulated for professional registration through the national allied health boards [3] (~20-30 hours per year = ~25-35 min per week for a full-time AHP). The authors believe this is insufficient and the inclusion of such a low minimum number of CPD hours in current standards may actually be reinforcing the understanding of many AHPs in this study that professional development is a separate formal activity away from direct clinical care and everyday work/learning. The determinants of how AHPs perceive workplace learning opportunities are likely to be many and complex (see Billett 2004 [4]). However, educating AHPs to improve their understanding of the benefits and value of workplace learning, the nature of workplace learning, in particular drawing attention to the often over-looked less formal learning, could be used as one strategy to improve their perceptions of, and motivation to more fully engage in and promote, workplace learning.

### Use of the COM-B model to interpret factors influencing workplace learning

This was the first study to use a model or system of behaviour, the ‘COM-B system’ [17], to help describe the barriers to, and enablers of, workplace learning. Through this model, barriers and enablers were reported as influencing workplace learning through affecting the capability, opportunity and/or motivation to engage in workplace learning (Figure 2). Use of this model facilitated interpretation of how barriers and enablers affected workplace learning, and importantly provides a framework to assist in the subsequent design of recommendations and implementation strategies to improve workplace learning. Of note however, is that (at least) for study of the behaviour of workplace learning, it is suggested that an additional interaction exists between opportunity and capability whereby factors that affected the opportunity to engage in workplace learning (e.g. management support) also affected the capability of AHPs (at an individual, team or system level) to engage in workplace learning. It is suggested that this interaction also exists in the opposite direction, such that factors that affected the capability of AHPs to engage in workplace learning (e.g. lack of dedicated professional educators) affected the opportunity for AHPs to engage in workplace learning. It is suggested that these interactions also be considered when designing intervention strategies to optimise workplace learning.

### Limitations

As with most research studies, this study had several limitations. First, the short timeline for recruitment and data collection meant that data saturation was not achieved. However the sampling methods used ensured the views of a broad cross-section of AHPs employed within the study setting were included and in the final focus groups, few new barriers and enablers arose, suggesting that the study may have been close to the point of data saturation.

Despite the use of maximum variation sampling some allied health professions were less represented than others, and some professions were not represented at all. This reflected not only the differences in size of the NSW Health workforce within individual allied health disciplines, but perhaps also the allied health professional structures within the study setting. While this may limit generalisability of the findings to groups less represented, there was evidence of many of the same barriers and enablers existing within all 10 allied health professions represented. Therefore it is likely that factors affecting workplace learning in other allied health professions not represented would include many of those reported here.

The use of a sample of volunteers meant participants were self-selected and therefore may have been more likely to have a greater understanding of, and/or interest in, workplace learning and/or have encountered more barriers to workplace learning than those who did not volunteer. However, a range of interpretations and understanding of workplace learning was evident among participants and a range of barriers and enablers were reported. In fact, it may have been that the extent of some barriers, such as ‘heavy workload’ or ‘lack of management support’ were not fully realised due to staff most affected by these barriers not having the time or not being supported to participate in this study. As for other studies employing interviews or focus groups, it must also be acknowledged that data collected and presented reflects ‘…accounts of phenomena, rather than any direct evidence of those phenomena.’ ([19] p.232) i.e. data reflects what AHPs said was occurring, rather than direct evidence (e.g. through observation) of what was occurring.

Finally, the range of individual interpretations of workplace learning may have also affected the findings. However, it is believed that by including a broad sample of participants, by intentionally not limiting participants in their definition of workplace learning and through framing probing questions in different ways, that data were not limited by individual interpretations of workplace learning. Rather, it is believed that the variation in individual interpretations of workplace learning added to the richness of data collected.

## Conclusion

To our knowledge this was the first study to specifically explore the concept of workplace learning in an allied health setting. The aim was to identify barriers to, and enablers of, workplace learning for AHPs employed within NSW Health. A key set of factors that enabled or restricted workplace learning through affecting the capability, opportunity and motivation of AHPs to engage in workplace learning were identified. These included access to peers, expertise and learning networks, clinical and administrative workload, management support for learning and staff attitudes toward learning. Identification of these barriers and enablers provides evidence for individuals, managers and organisations to inform specific strategies to enhance learning opportunities. The findings also challenge existing perceptions and attitudes of professional development of many allied health staff, in particular the important role played by workplace learning in addition to courses and conferences outside of the workplace.

This study raises important questions about how organisations can support workplace learning for AHPs more effectively. This could include actively promoting the creation of peer/clinical networks across organisational and geographical boundaries, introducing targeted education programs for managers on how to effectively facilitate workplace learning, reallocation of funding for the creation of additional dedicated education roles to support time poor clinicians and managers and enhancing available learning resources such as investment in technology. Future research is needed to evaluate the effectiveness of such strategies across a range of settings.

Learning opportunities which are geared toward workplace activity have the potential to improve outcomes for patients, staff and the organisation as a whole. While attendance at external courses and conferences remains an important component of professional development for AHPs, this can be enriched by valuing and seeking out learning experiences that occur within the workplace.

## Competing interests

The authors declare that they have no competing interests.

## Authors’ contributions

BL contributed to study design, collected and analysed the data and drafted the manuscript. DP conceptualised and planned the study and assisted with study design, data collection, data analysis and manuscript preparation. JD conceptualised and planned the study and assisted with study design and manuscript preparation. GH assisted with planning the study and advised on study design, data collection and data analysis. DS advised on study design, data collection and data analysis and assisted with manuscript preparation. AM provided guidance with study design, data collection and data analysis and assisted with and provided oversight for drafting the manuscript. All authors read and approved the final manuscript.

## Authors’ information

BL is a qualified physiotherapist and exercise physiologist. He has worked as a physiotherapy clinician and researcher within NSW Health, and was employed by HETI to carry out this study. His research background is predominantly in quantitative research. Because of his previous employment as an allied health clinician within NSW Health some participants recruited to the study were known to him.

DP is a Senior Program Officer at HETI, and has a clinical background in social work. She has a Masters in Couple and Family Therapy and was previously employed within NSW Health as a social worker.

JD is a Senior Program Officer at HETI, and has a clinical background in occupational therapy. She has a Certificate IV in Workplace Assessment and Training and a Masters in Health Management and was previously employed within NSW Health as an occupational therapist.

GH is Deputy Chief Executive at the Health Education and Training Institute. She has a PhD in medicine, and has experience in mixed-methods research. She has previously held senior academic positions at the University of Newcastle and The University of Sydney and has taught research methods to health and medical students and practicing clinicians including AHPs.

DS has qualifications in physiotherapy and clinical education. He is employed as Senior Program Officer with HETI’s Rural Research Capacity Building Program.

AM is an occupational therapist, a health services researcher and a senior lecturer at The University of Sydney. She has experience conducting qualitative research as well as randomised controlled trials.

## Pre-publication history

The pre-publication history for this paper can be accessed here:

http://www.biomedcentral.com/1472-6963/14/134/prepub
